# Safety and feasibility of peripherally inserted central catheter (PICC) placement in patients with newly diagnosed acute leukemia: A single-center retrospective study

**DOI:** 10.1007/s00277-026-07241-4

**Published:** 2026-08-12

**Authors:** Yulei Wei, Xiaohong Zhang, Fei Zhao, Yang He, Jinlian Hu

**Affiliations:** Shanghai Institute of Hematology, State Key Laboratory of Medical Genomics, National Research Center for Translational Medicine at Shanghai, Ruijin Hospital, Shanghai Jiao Tong University School of Medicine, Shanghai, 200025 China

**Keywords:** Peripherally inserted central catheter, Acute leukemia, Thrombocytopenia, Fibrinogen, Bleeding risk prediction

## Abstract

Patients with newly diagnosed acute leukemia are at increased risk of bleeding during vascular access because of thrombocytopenia and coagulopathy. The primary objective of this single-center retrospective study (November 2023–August 2024; Ruijin Hospital) was to evaluate the feasibility and safety of a peripherally inserted central catheter (PICC) puncture and nursing protocol incorporating ultrasound-guided Seldinger puncture, controlled sheath withdrawal with gauze-assisted compression, and localized hemostatic dressing in 101 patients prior to chemotherapy. The first-attempt success rate was 95.1%. At 24 h, active oozing occurred in 14 patients (13.9%); all events were CTCAE grade 1–2 and were controlled with local measures. Platelet count (PLT; OR = 0.972, *P* = 0.029) and fibrinogen (Fg; OR = 0.594, *P* = 0.049) were independent predictors of bleeding. On ROC analysis, PLT showed greater predictive value (AUC = 0.762, 95% CI 0.628–0.880). Catheter malposition and thrombosis each occurred in 1.0% of patients (CTCAE grade 2 and grade 1, respectively); no phlebitis, catheter-related infection, or grade ≥ 3 adverse events were reported. The technique was safe and feasible, and PLT and Fg helped predict bleeding risk. Prospective multicenter validation is warranted.

**Supplementary Information:**

The online version contains supplementary material available at https://doi.org/10.1007/s00277-026-07241-4.

## Introduction

Acute leukemia is an aggressive hematologic malignancy with rapid onset and pronounced clinical manifestations, and its annual worldwide incidence is approximately 3–5 per 100,000 population. Chemotherapy remains the cornerstone of treatment and has substantially improved patient outcomes. Timely induction therapy and adequate supportive care reduce early mortality, and long-term survival has improved markedly compared with the pre-chemotherapy era [1, 2]. Despite these advances, approximately 10–20% of patients still die within the first month after diagnosis, so timely and effective chemotherapy remains essential [3]. During treatment, patients frequently develop hematologic and coagulation abnormalities that compromise chemotherapy delivery (by limiting drug tolerance and delaying treatment schedules) and increase the risk of serious complications such as hemorrhage, infection, and thrombosis [4]. Bleeding in these patients results mainly from thrombocytopenia caused by leukemic infiltration of the bone marrow and suppression of normal hematopoiesis; the same disturbances also increase the risk of thrombotic and infectious complications, and both adversely affect prognosis [4, 5].

Chemotherapy is commonly administered through peripherally inserted central catheters (PICCs). Because coagulation abnormalities and immunosuppression are common in acute leukemia, these patients are particularly susceptible to catheter-related infection, bleeding, and thrombosis [6]. Conventional PICC insertion techniques, which rely on standard vein puncture and manual hemostasis, are poorly suited to patients with acute leukemia, who frequently have thrombocytopenia (mean platelet counts of 20–70 × 10⁹/L in published studies) and prolonged coagulation times [4, 7]. These abnormalities contribute to fragile veins, poor tissue elasticity, and difficult hemostasis, often leading to prolonged bleeding or hematoma formation after catheter placement [8–10]. For example, puncture-site oozing has been reported in up to 24% of leukemia patients undergoing standard PICC insertion [8]; techniques tailored to this high-risk population are needed.

Several prospective and retrospective studies have specifically evaluated PICCs in patients with acute leukemia undergoing induction chemotherapy. In a randomized comparison, Picardi et al. showed that first-line PICC placement for remission-induction chemotherapy in acute myeloid leukemia was feasible and had a better safety profile than centrally inserted central venous catheters (36-percentage-point reduction in the composite rate of catheter-related bloodstream infection and venous thrombosis; relative risk 0.266, *P* = 0.0003) [11]. A subsequent single-center retrospective study of intravascular complications by insertion site found a significantly lower risk of catheter-related symptomatic deep-vein thrombosis and bloodstream infection with peripherally inserted than with centrally inserted catheters [12]. In a later report, the same group described real-world experience showing that PICCs can be safely placed and maintained in patients with acute leukemia who develop severe thrombocytopenia during remission-induction chemotherapy; in that series, grade ≥ 3 bleeding complications (hematoma or arterial puncture) were significantly less frequent with peripherally inserted than with centrally inserted catheters (3 events in the basilic/brachial cohort vs. 10 and 12 in the subclavian and internal jugular cohorts, respectively; *P* = 0.015) [13].

Persistently high early mortality makes safe and effective catheterization an essential part of supportive care throughout treatment [14]. Close monitoring of blood counts and coagulation parameters is routine during treatment [6]. PICC-related complications remain common in patients with hematologic malignancies, with higher rates of catheter-associated infection and thrombosis than in solid-tumor populations [8, 9]. Although technological improvements have made catheters safer overall, evidence on device modifications or insertion techniques designed for patients with thrombocytopenia or coagulation abnormalities remains scarce [10, 15]. In contrast to conventional PICC insertion, the protocol evaluated here combines ultrasound-guided puncture, atraumatic catheter advancement exploiting skin elasticity, controlled sheath withdrawal with immediate manual compression, and a risk-adapted hemostatic dressing and compression regimen; the rationale for each element is detailed in the Methods. We therefore conducted this single-center retrospective study to evaluate the safety and feasibility of this standardized PICC puncture and nursing protocol in patients with newly diagnosed acute leukemia. The primary outcomes were procedural success and early catheter-related complications (with emphasis on bleeding control and infection prevention); secondary analyses examined pre-procedural predictors of puncture-site bleeding.

## Materials and methods

### Study design and patients

The primary objective of this single-center retrospective study was to evaluate the feasibility (procedural success) and safety (catheter-related complications, graded according to CTCAE v4.03) of a standardized PICC puncture and nursing protocol in patients with newly diagnosed acute leukemia; a secondary objective was to identify pre-procedural predictors of puncture-site bleeding. The study included patients with acute leukemia admitted to the Department of Hematology, Ruijin Hospital, Shanghai Jiao Tong University School of Medicine, between November 2023 and August 2024. The inclusion criteria were as follows: (1) a diagnosis of acute leukemia (acute lymphoblastic leukemia [ALL] or acute myeloid leukemia [AML]) according to the 2016 WHO criteria [16], with no prior intravenous chemotherapy; (2) age of 14 years or older; and (3) PICC placement during hospitalization. The 2016 WHO criteria were used because they were the standard diagnostic framework in routine clinical use at our center throughout the study period; for AML and ALL they are largely concordant with the 2022 (5th edition) WHO classification and the 2022 International Consensus Classification, so this choice is unlikely to have materially affected patient selection or the study findings. All consecutive eligible patients admitted during this period were enrolled; the final cohort (101 patients: 17 with ALL and 84 with AML) was therefore determined by the study time window rather than by a prespecified sample-size calculation.

The exclusion criteria were: (1) incomplete medical or nursing documentation of PICC insertion or follow-up; (2) pre-existing venous thrombosis or a history of major upper-limb vascular surgery before the current admission; and (3) hemodynamic instability or other life-threatening comorbidities during the PICC dwell period that precluded routine catheter care. Patients were further stratified into high-, intermediate-, and low-risk groups according to the 2022 European LeukemiaNet (ELN) risk classification for AML [17] and the 2023 National Comprehensive Cancer Network (NCCN) clinicogenetic risk criteria for ALL [18]. This stratification refers to disease-related prognostic risk (i.e., cytogenetic and molecular risk of treatment resistance and relapse), not to bleeding or procedural risk; it was used descriptively to compare baseline characteristics and complication rates across prognostic subgroups.

The study was approved by the Ethics Committee of Ruijin Hospital, Shanghai Jiao Tong University School of Medicine [(2025) Clinical Ethics Approval No. (208)]. The requirement for informed consent was waived because of the retrospective nature of the study.

### PICC puncture and nursing protocol

All patients were managed according to a standardized PICC puncture and nursing protocol. The medical history was reviewed, contraindications were excluded, and laboratory indices were assessed and used to identify patients at increased risk of puncture-site bleeding (platelet count [PLT] ≤ 20 × 10⁹/L or fibrinogen [Fg] ≤ 1.8 g/L), who received the risk-adapted compression regimen described below. The purpose of the procedure and the cooperation required were explained to patients and their families, and informed consent was obtained. All patients underwent ultrasound-guided modified Seldinger PICC placement with a single-lumen 4-Fr three-way valve catheter (Bard, USA) [19]. All procedures were performed by certified nurses with more than 10 years of experience. Skin incision, guidewire advancement, sheath insertion, and catheter placement were performed with gentle rotation, taking advantage of skin elasticity to minimize trauma and bleeding. During sheath removal, pressure was applied with the left palm to control bleeding. Topical hemostatic agents were applied, and the puncture site was dressed with gelatin sponge, layered gauze, and a transparent adhesive film to secure the catheter and maintain hemostasis. The detailed step-by-step protocol followed during PICC implantation and post-procedural care is provided in the Supplementary Material.

Differences from conventional practice and rationale. Compared with conventional PICC insertion, which generally relies on standard vein puncture followed by manual compression for hemostasis, the present protocol incorporated the following technical refinements, each directed at the specific vulnerabilities of patients with acute leukemia (thrombocytopenia, coagulopathy, fragile veins, and poor tissue elasticity): (1) routine ultrasound guidance for vein selection and puncture, to minimize repeated puncture attempts and inadvertent vessel-wall injury; (2) gentle rotational advancement of the guidewire, peel-away sheath, and catheter, exploiting skin elasticity to reduce mechanical trauma to fragile tissues; (3) controlled sheath withdrawal with immediate pressure applied with the left palm, to prevent back-bleeding along the sheath tract during the step at which bleeding risk is greatest; (4) application of topical hemostatic agents and a layered dressing (gelatin sponge, layered gauze, and transparent adhesive film) to provide sustained localized hemostasis without systemic pro-coagulant therapy; and (5) a risk-adapted compression regimen (semi-roll spiral elastic bandage for 20 min) reserved for patients with PLT ≤ 20 × 10⁹/L or Fg ≤ 1.8 g/L, in whom puncture-site bleeding risk is highest. These refinements make up a standardized, reproducible insertion and nursing protocol rather than a new catheter device.

Patients with PLT ≤ 20 × 10⁹/L or Fg ≤ 1.8 g/L received semi-roll spiral compression with an elastic bandage for 20 min. Limb movement was restricted on the day of puncture, and heat application was avoided. If active bleeding occurred, the dressing was changed and compression was reapplied for another 20 min.

### Data collection

Baseline demographic, clinical, and laboratory data (age, sex, leukemia type, laboratory indices, and puncture site) were collected retrospectively from the medical records. No patient received platelet concentrate, fresh frozen plasma, or cryoprecipitate within 48 h before PICC insertion; all laboratory indices therefore represented untreated baseline values. Catheterization outcomes included successful single-attempt puncture (no needle retraction or change of puncture site) and successful single-attempt catheter placement (appropriate tip position on chest radiography). Catheter-related complications included persistent oozing (bleeding within 24–72 h after catheterization, assessed at 24 h), phlebitis (redness, swelling, pain, or cord formation), catheter malposition (tip outside the target area), and catheter-associated thrombosis (thrombus at or within the catheter tip). All complications were graded according to the National Cancer Institute Common Terminology Criteria for Adverse Events (CTCAE), version 4.03: puncture-site bleeding was graded with the hemorrhage scale, catheter malposition with “vascular access complication,” catheter-associated thrombosis with “thromboembolic event,” and catheter-related infection with “catheter related infection”.

### Statistical analysis

All data were analyzed using SPSS version 26.0 (IBM Corp., Armonk, NY, USA). The normality of quantitative variables was assessed with the Shapiro–Wilk test. Normally distributed variables were expressed as mean ± standard deviation (range) and compared across the three risk groups using one-way ANOVA, or Welch ANOVA when the assumption of homogeneity of variance was violated; skewed variables were compared using the Kruskal–Wallis test. Between-group comparisons of bleeding and non-bleeding patients were performed using the independent-samples t-test or the Mann–Whitney U test, as appropriate. Categorical variables were expressed as frequencies (percentages) and compared using the chi-square test; Fisher’s exact test was used when any expected cell frequency was below 5. Univariable and multivariable logistic regression analyses were performed to identify variables associated with post-puncture bleeding, and their predictive performance was evaluated by receiver operating characteristic (ROC) curve analysis with the area under the curve (AUC). A two-sided *P* < 0.05 was considered statistically significant.

## Results

### Patient characteristics

The study cohort comprised 101 patients (55 males and 46 females) aged 15–74 years (mean age, 47.31 ± 16.59 years). Seventeen patients (16.83%) had ALL and 84 (83.17%) had AML. By risk stratification, 43 patients (42.57%) were high-risk, 23 (22.77%) intermediate-risk, and 35 (34.65%) low-risk. Baseline laboratory values included WBC 21.53 ± 34.63 × 10⁹/L, RBC 2.55 ± 0.87 × 10¹²/L, hemoglobin 82.59 ± 24.36 g/L, PLT 69.60 ± 65.89 × 10⁹/L, Fg 3.17 ± 1.58 g/L, PT 13.42 ± 1.69 s, APTT 29.16 ± 3.29 s, TT 17.83 ± 1.70 s, and D-dimer 4.64 ± 7.24 mg/L. None of the patients received prophylactic transfusion before catheter insertion. Puncture sites were predominantly the right basilic vein (83.20%), followed by the right brachial vein (10.90%), the left basilic vein (4.90%), and the right cephalic vein (1.00%). Age and the laboratory parameters WBC, PLT, PT, and D-dimer differed significantly among the risk groups (all *P* < 0.05), whereas sex, educational level, diagnosis, puncture site, and the remaining laboratory parameters (RBC, Hb, Fg, TT, and APTT) did not (Table 1).

**Table 1:** Patient characteristics by risk group

Characteristics	Overall (*n* = 101)	High risk (*n* = 43)	Intermediate risk (*n* = 23)	Low risk (*n* = 35)	*P*
Sex, n (%)					0.499
Male	55 (54.50%)	22 (51.20%)	15 (65.20%)	18 (51.50%)	
Female	46 (45.50%)	21 (48.80%)	8 (34.80%)	17 (48.50%)	
Age, mean ± SD (range)	47.31 ± 16.59 (15–74)	41.93 ± 16.73 (15–72)	48.22 ± 15.29 (17–72)	53.31 ± 15.42 (25–74)	0.012
Educational level, n (%)					0.252
Basic Education	61 (60.40%)	22 (51.20%)	16 (69.50%)	23 (65.80%)	
Higher Education	40 (39.60%)	21 (48.80%)	7 (30.50%)	12 (34.20%)	
Diagnosis, n (%)					0.780
ALL	17 (16.83%)	7 (16.30%)	3 (13.10%)	7 (20.00%)	
AML	84 (83.17%)	36 (83.70%)	20 (86.90%)	28 (80.00%)	
Laboratory findings, mean ± SD (range)					
WBC, ×10⁹/L	21.53 ± 34.63 (0.40–223.20)	45.81 ± 42.39 (10.40–223.20)	4.04 ± 3.07 (0.40–9.48)	3.19 ± 1.90 (0.69–8.75)	< 0.001
RBC, ×10¹²/L	2.55 ± 0.87 (0.38–5.17)	2.67 ± 1.05 (0.38–5.17)	2.33 ± 0.67 (1.04–3.74)	2.56 ± 0.70 (1.33–4.39)	0.271
Hb, g/L	82.59 ± 24.36 (37.00–150.00)	84.26 ± 27.31 (37.00–150.00)	75.17 ± 20.49 (38.00–124.00)	85.43 ± 22.46 (43.00–141.00)	0.248
PLT, ×10⁹/L	69.60 ± 65.89 (7.00–380.00)	54.88 ± 47.46 (7.00–258.00)	23.48 ± 7.56 (10.00–36.00)	118.00 ± 76.31 (41.00–380.00)	< 0.001
TT, s	17.83 ± 1.70 (13.90–26.50)	17.93 ± 2.13 (13.90–26.50)	17.78 ± 1.58 (15.50–21.30)	17.74 ± 1.10 (15.40–19.70)	0.843
PT, s	13.42 ± 1.69 (10.00–19.40)	14.04 ± 1.64 (11.10–19.10)	13.48 ± 1.95 (11.20–19.40)	12.62 ± 1.19 (10.00–15.60)	< 0.001
Fg, g/L	3.17 ± 1.58 (0.80–9.00)	2.92 ± 1.64 (0.80–9.00)	3.37 ± 1.47 (0.90–6.30)	3.36 ± 1.59 (1.40–8.70)	0.237
D-Dimer, mg/L	4.64 ± 7.24 (0.19–40.00)	6.61 ± 8.73 (0.40–40.00)	3.45 ± 4.49 (0.51–18.95)	3.01 ± 6.19 (0.19–33.74)	0.001
APTT, s	29.16 ± 3.29 (22.50–38.60)	29.48 ± 3.85 (22.50–38.60)	29.44 ± 3.36 (25.00–36.80)	28.57 ± 2.37 (25.80–33.80)	0.692
Puncture site, n (%)					0.570
Right Basilic Vein	84 (83.20%)	36 (83.70%)	18 (78.30%)	30 (85.80%)	
Right Brachial Vein	11 (10.90%)	6 (13.90%)	3 (13.10%)	2 (5.70%)	
Right Cephalic Vein	1 (1.00%)	0 (0.00%)	0 (0.00%)	1 (2.80%)	
Left Basilic Vein	5 (4.90%)	1 (2.40%)	2 (8.60%)	2 (5.70%)	

*P* values were derived from one-way ANOVA (Hb), Welch ANOVA (RBC), the Kruskal–Wallis test (Age, WBC, PLT, TT, PT, Fg, D-dimer, APTT), the chi-square test (Sex, Educational level, Diagnosis), or Fisher’s exact test (Puncture site), as appropriate

### Catheterization outcomes

All 101 patients underwent successful PICC placement before intravenous chemotherapy. First-attempt puncture was successful in 96 patients (95.1%); 5 patients required adjustment of the puncture site.

### Complications

Catheter-related complications, graded according to CTCAE v4.03, are summarized in Table 2. At 24 h after catheterization, 14 patients (13.9%) had active oozing at the insertion site; the rate did not differ significantly across the high-risk (6/43, 13.9%), intermediate-risk (5/23, 21.7%), and low-risk (3/35, 8.5%) groups (*P* = 0.349, Fisher’s exact test). All 14 events were grade 1 (*n* = 8) or grade 2 (*n* = 6) and were controlled with local compression and dressing changes; none required transfusion or radiologic, endoscopic, or operative intervention (grade 3), and no event was life-threatening (grade 4) or fatal (grade 5). During induction therapy, one high-risk patient (1.0% overall) developed catheter malposition (vascular access complication, grade 2), which was corrected by ultrasound-guided repositioning, and one low-risk patient (1.0% overall) developed an asymptomatic peri-catheter thrombosis (thromboembolic event, grade 1), detected incidentally on ultrasound and managed conservatively. No phlebitis or catheter-related infection was observed in any risk group. Overall, no grade 3–5 catheter-related adverse events occurred (Table 2).

**Table 2:** Catheter-related complications in 101 patients with acute leukemia, graded according to CTCAE v4.03

Complication (CTCAE v4.03 term)	Overall (*n* = 101), n (%)	Grade 1, *n*	Grade 2, *n*	Grade 3–5, *n*	Low risk (*n* = 35), n (%)	Intermediate risk (*n* = 23), n (%)	High risk (*n* = 43), n (%)	*P*
Puncture-site bleeding/oozing (hemorrhage)^a^	14 (13.9)	8	6	0	3 (8.5)	5 (21.7)	6 (13.9)	0.349
Catheter malposition (vascular access complication)^b^	1 (1.0)	0	1	0	0	0	1 (2.3)	/
Catheter-associated thrombosis (thromboembolic event)^c^	1 (1.0)	1	0	0	1 (2.8)	0	0	/
Phlebitis	0	0	0	0	0	0	0	/
Catheter-related infection^d^	0	0	0	0	0	0	0	/

^a^CTCAE v4.03 hemorrhage scale: grade 1 = mild, intervention not indicated; grade 2 = moderate symptoms, medical or local intervention indicated; grade 3 = transfusion, radiologic, endoscopic, or elective operative intervention indicated; grade 4 = life-threatening consequences, urgent intervention indicated; grade 5 = death

^b^CTCAE v4.03 “Vascular access complication”: grade 2 = device dislodgement, blockage, leak, or malposition; device replacement indicated

^c^CTCAE v4.03 “Thromboembolic event”: grade 1 = venous thrombosis (e.g., superficial thrombosis); grade 2 = uncomplicated deep-vein thrombosis, medical intervention indicated

^d^CTCAE v4.03 “Catheter related infection”: grade 2 = localized, local and/or oral intervention indicated; grade 3 = IV antimicrobial, radiologic, or operative intervention indicated

CTCAE = Common Terminology Criteria for Adverse Events. No grade 3–5 events were observed

### Univariable and multivariable analysis

**Fig. 1:**
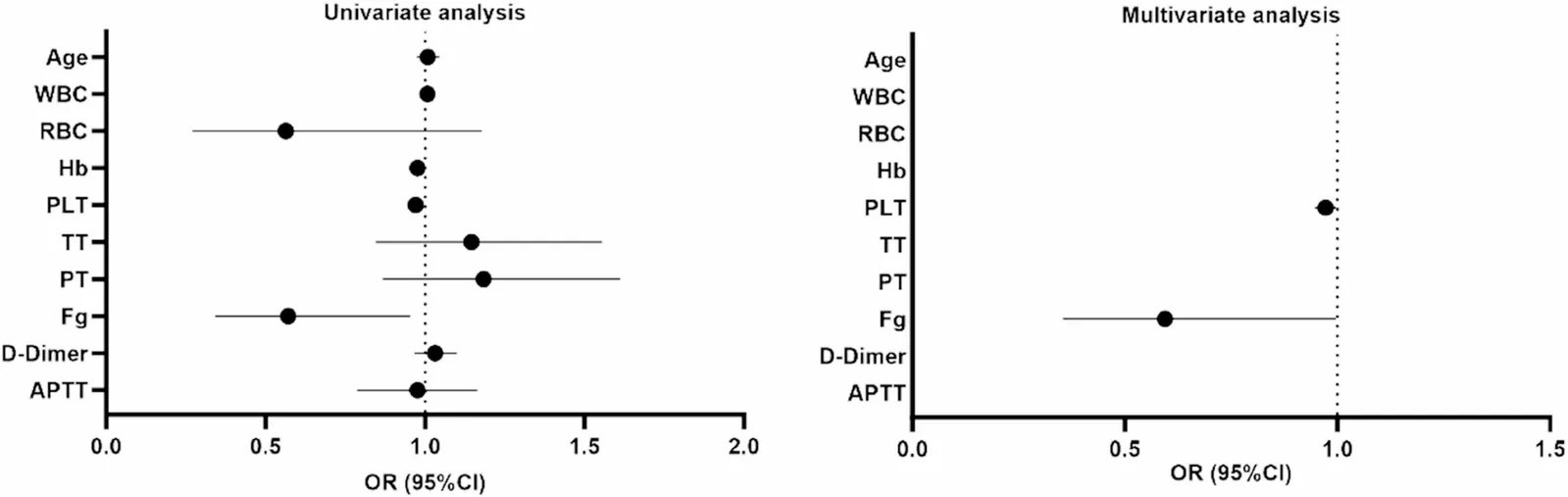
Multivariable logistic regression analysis of hematological variables associated with post-puncture bleeding. The forest plot shows effect sizes and 95% CIs

On univariable logistic regression, PLT and Fg levels were associated with active oozing (PLT: OR 0.970, 95% CI 0.945–0.994, *P* = 0.023; Fg: OR 0.570, 95% CI 0.341–0.953, *P* = 0.032). Both variables remained independently associated with active oozing in the multivariable analysis, with little attenuation of the effect estimates (PLT: OR 0.972, 95% CI 0.947–0.997, *P* = 0.029; Fg: OR 0.594, 95% CI 0.354–0.997, *P* = 0.049) (Fig. 1; Table 3).

**Table 3:** Multivariable logistic regression analysis of hematological variables associated with post-puncture bleeding

Variables	Univariable analysis		Multivariable analysis	
	OR (95% CI)	*P*	OR (95% CI)	*P*
Age	1.008 (0.973–1.044)	0.667	-	-
WBC	1.007 (0.993–1.021)	0.302	-	-
RBC	0.563 (0.269–1.178)	0.128	-	-
Hb	0.976 (0.950–1.003)	0.082	-	-
PLT	0.970 (0.945–0.994)	0.023	0.972 (0.947–0.997)	0.029
TT	1.145 (0.844–1.555)	0.385	-	-
PT	1.183 (0.868–1.612)	0.286	-	-
Fg	0.570 (0.341–0.953)	0.032	0.594 (0.354–0.997)	0.049
D-Dimer	1.031 (0.966–1.099)	0.363	-	-
APTT	0.976 (0.818–1.163)	0.785	-	-

### Group comparisons

**Fig. 2:**
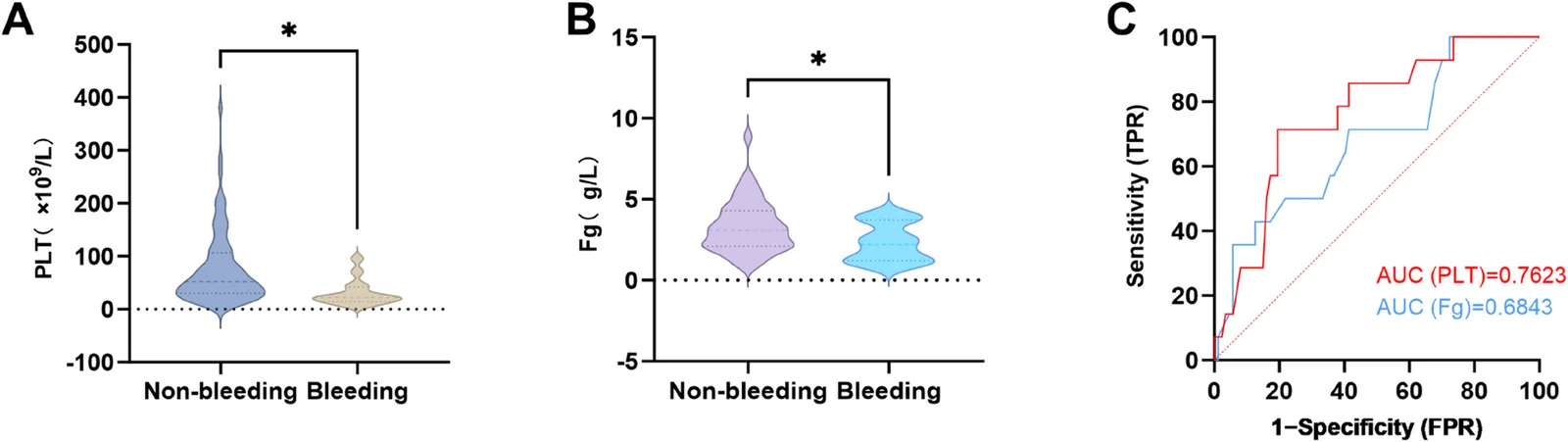
Differences in PLT and Fg levels between non-bleeding and bleeding groups. **A** Violin plots of PLT distribution. **B** Violin plots of Fg levels. (*P < 0.05, Mann–Whitney U test). **C** ROC curve analysis of PLT and Fg predicting post-puncture bleeding

Median platelet counts and fibrinogen levels were significantly higher in the non-bleeding group than in the bleeding group (PLT: 52.00 [30.00–102.00] vs. 22.00 [16.50–37.00] × 10⁹/L, *P* = 0.002; Fg: 3.10 [2.15–4.30] vs. 2.20 [1.23–3.45] g/L, *P* = 0.028) (Fig. 2A and B).

### Predictive performance

On ROC analysis, PLT (AUC = 0.762, 95% CI 0.628–0.880) showed greater predictive value than Fg (AUC = 0.684, 95% CI 0.523–0.829); the optimal PLT cutoff was 25 × 10⁹/L (sensitivity 71.4%, specificity 80.5%) (Fig. 2C).

## Discussion

This study evaluated the safety, feasibility, and early complications of a PICC puncture and nursing protocol in patients with newly diagnosed acute leukemia. The technique achieved a high first-attempt success rate (95.1%) and low early complication rates, and PLT and Fg were independent predictors of post-puncture oozing. These data give clinicians concrete parameters for pre-procedural bleeding-risk assessment when establishing central venous access before induction chemotherapy.

PICC use has grown steadily in leukemia care because these catheters are minimally invasive and provide durable venous access throughout chemotherapy [20]. Nonetheless, patients with acute leukemia are more prone to puncture-site oozing and hematoma formation than those with solid tumors because of severe thrombocytopenia and the coagulation deficits of bone marrow failure [7, 8]. Most previous work has focused on post-placement nursing care rather than on the insertion procedure itself. We therefore report procedural outcomes and early complications in 101 consecutive patients, with emphasis on bleeding risk factors.

The first-attempt success rate in this study (95.1%) is comparable to that reported by specialized oncology centers [9] and slightly higher than the 85–90% reported in general medical populations [21]. This difference may reflect operator experience, which has been shown to influence catheterization outcomes [22]. In our cohort, most PICCs were inserted through the right basilic vein, which offers a relatively straight course and is generally preferred for catheter stability.

Only 13.9% of our patients had active oozing at 24 h, versus 20–25% in previous studies of leukemia or mixed cancer populations [10, 23]. This difference is clinically meaningful and may partly reflect the technique itself: careful puncture-angle control, standardized vein selection, and a uniform compression protocol have each been associated with fewer procedural complications in patients with hematologic malignancies [24, 25]. Catheter malposition and thrombosis rates were also low (1.0% each), and no phlebitis occurred, figures at the lower end of the range reported in oncology-specific and general PICC studies [10, 23]. Catheter insertion was completed without prophylactic platelet transfusion even in patients with severe thrombocytopenia (platelet count as low as 7 × 10⁹/L), and all bleeding events were limited to CTCAE grade 1–2, supporting the feasibility of PICC placement without routine prophylactic transfusion in this population.

Compared with previous studies specifically addressing PICCs in acute leukemia, the pattern of catheter-related complications in our cohort was dominated by early puncture-site oozing, whereas thrombotic and infectious events were rare. Picardi et al. reported that, during remission-induction chemotherapy, peripherally inserted catheters carried a significantly lower incidence of the composite of catheter-related bloodstream infection and venous thrombosis than centrally inserted catheters in a randomized trial [11], and a significantly lower risk of symptomatic deep-vein thrombosis and bloodstream infection in a subsequent larger retrospective analysis [12]. PICCs could also be safely managed in patients who developed severe thrombocytopenia [13]. Consistent with these reports, our series included only one asymptomatic peri-catheter thrombosis (1.0%, CTCAE grade 1) and no phlebitis or catheter-related bloodstream infection. This low thrombotic rate agrees with that reported for PICCs in acute myeloid leukemia (symptomatic venous thrombosis, 2.1%, in the randomized trial by Picardi et al. [11]) and is lower than the PICC-related symptomatic venous thrombosis rate reported in classical Hodgkin lymphoma (4.5%) [26], although that population differs in disease biology, thrombotic risk profile, and catheter dwell time (median 300 days in that series). In contrast to those studies, in which thrombosis and infection were the principal concerns, insertion-site bleeding was the dominant early complication in our cohort, although all events were CTCAE grade 1–2 and were controlled with local measures. For patients with newly diagnosed acute leukemia complicated by thrombocytopenia and coagulopathy, pre-procedural risk stratification based on PLT and Fg therefore seems warranted.

PLT and Fg levels independently predicted puncture-site oozing in our cohort. This observation fits the pathophysiology of acute leukemia, in which impaired platelet production and consumptive coagulopathy increase bleeding susceptibility. Because no patient received transfusion before insertion, the platelet and fibrinogen values analyzed here reflect untreated bone marrow failure rather than post-transfusion levels; this removes an important source of confounding in the association with puncture-site bleeding. Previous studies have similarly identified thrombocytopenia and hypofibrinogenemia as major determinants of bleeding risk during invasive procedures [15, 21, 27]. In contrast, PT, APTT, and D-dimer were not predictive in our analysis, which differs from reports proposing a broader role for conventional coagulation tests in bleeding-risk stratification [28]. This discrepancy likely reflects our cohort’s relatively stable baseline coagulation status and the standardized technique, which minimizes mechanical trauma.

On ROC analysis, PLT outperformed Fg (AUC 0.76 vs. 0.68). Platelet count was thus the more practical single parameter for pre-insertion risk assessment, in line with recommendations to assess both platelet and fibrinogen levels in at-risk hematology patients [27].

This study has several limitations. Without a control group, we cannot conclude that this technique is superior to standard PICC insertion; the study was designed around safety and feasibility, and its single-center retrospective design with 101 patients limits generalizability. All procedures were also performed by highly experienced nurses, so outcomes may differ in centers with less experienced staff. Finally, follow-up covered only early complications; longer-term outcomes (late infection, catheter removal, patient comfort, and cost) and advanced coagulation monitoring such as thromboelastography were not assessed.

In conclusion, this PICC puncture and nursing protocol was safe and feasible in newly diagnosed acute leukemia, achieving a 95.1% first-attempt success rate with only grade 1–2 early complications. Pre-procedural PLT and Fg levels helped identify patients at risk of puncture-site bleeding and may guide planning; prospective multicenter validation is warranted.

## Supplementary Information

Below is the link to the electronic supplementary material.

Supplementary Material 1

## Data Availability

The authors confirm that the data supporting the findings of this study are available within the article.
