# Supplementary material for: Safety and feasibility of peripherally inserted central catheter (PICC) placement in patients with newly diagnosed acute leukemia: A single-center retrospective study
Source: Ann Hematol. 2026 Aug 12;105(12):505. doi: 10.1007/s00277-026-07241-4 (PMC13748009; doi:10.1007/s00277-026-07241-4)
Supplement: Supplementary file 1 [file 277_2026_7241_MOESM1_ESM.docx]

**Supplementary Material**

**Detailed Step-by-Step Protocol for PICC Implantation and Post-Procedural Nursing Care in Patients with Newly Diagnosed Acute Leukemia**

*Supplementary to: "Safety and Feasibility of Peripherally Inserted Central Catheter (PICC) Placement in Patients with Newly Diagnosed Acute Leukemia: A Single-Center Retrospective Study"*

# **1. Pre-procedural assessment**

**Step 1. Medical history review.** The medical history was reviewed for every patient, with particular attention to previous venous thrombosis, major upper-limb vascular surgery, bleeding history, and current anticoagulant or antiplatelet therapy. Patients with pre-existing venous thrombosis, a history of major upper-limb vascular surgery, or hemodynamic instability precluding routine catheter care were excluded from PICC placement under this protocol.

**Step 2. Laboratory assessment.** Baseline laboratory indices were obtained before the procedure: platelet count (PLT), fibrinogen (Fg), prothrombin time (PT), activated partial thromboplastin time (APTT), thrombin time (TT), and D-dimer. No patient received platelet concentrate, fresh frozen plasma, or cryoprecipitate within 48 hours before insertion, so all values represented untreated baseline levels.

**Step 3. Bleeding-risk identification.** Patients with PLT <= 20 x 10^9/L or Fg <= 1.8 g/L were identified as being at increased risk of puncture-site bleeding and were assigned to the risk-adapted compression regimen described in Section 5.

**Step 4. Ultrasound vein evaluation.** The veins of both upper extremities were evaluated by ultrasound before puncture. The basilic vein was preferred because of its relatively straight course; the brachial and cephalic veins were considered as alternatives. Vein diameter, depth, patency, and the absence of thrombus were confirmed.

# **2. Patient information and consent**

**Step 5. Explanation and consent.** The purpose of the procedure, the expected course of catheterization, possible complications (bleeding, infection, thrombosis, malposition), and the cooperation required during and after placement were explained to patients and their families. Written informed consent for the procedure was obtained in all cases.

# **3. Personnel and equipment**

**Step 6. Operators.** All procedures were performed by certified nurses with more than 10 years of experience in PICC placement, assisted according to the routine of the vascular access team.

**Step 7. Catheter and materials.** A single-lumen 4-Fr three-way valve catheter (Bard, USA) was used in all patients [1]. Standard materials included an ultrasound system with a vascular probe, a micropuncture/modified Seldinger set, sterile barrier precautions (cap, mask, sterile gown and gloves, large sterile drape), topical hemostatic agents, absorbable gelatin sponge, layered sterile gauze, a transparent adhesive film dressing, and a semi-roll spiral elastic bandage.

# **4. Insertion procedure**

**Step 8. Positioning and skin preparation.** The patient was placed supine with the arm abducted. The puncture area was disinfected widely, and maximal sterile barrier precautions were applied.

**Step 9. Ultrasound-guided puncture.** Under real-time ultrasound guidance, the selected vein was punctured using the modified Seldinger technique [1]. Routine ultrasound guidance was used for vein selection and puncture to minimize repeated puncture attempts and inadvertent vessel-wall injury.

**Step 10. Guidewire, sheath, and catheter advancement.** Skin incision, guidewire advancement, peel-away sheath insertion, and catheter placement were all performed with gentle rotation, taking advantage of skin elasticity to reduce mechanical trauma to fragile tissues and minimize bleeding.

**Step 11. Controlled sheath withdrawal.** During removal of the peel-away sheath - the step at which bleeding risk is greatest - immediate pressure was applied over the puncture site with the left palm to prevent back-bleeding along the sheath tract, while the catheter was secured with the other hand.

**Step 12. Catheter confirmation and flushing.** Blood return was confirmed, and the catheter was flushed and locked according to routine practice before dressing.

# **5. Hemostasis, dressing, and risk-adapted compression**

**Step 13. Localized hemostatic dressing.** Topical hemostatic agents were applied to the puncture site, which was then dressed with absorbable gelatin sponge covered by layered sterile gauze and a transparent adhesive film, to secure the catheter and provide sustained localized hemostasis without systemic pro-coagulant therapy.

**Step 14. Risk-adapted compression.** Patients identified in Step 3 (PLT <= 20 x 10^9/L or Fg <= 1.8 g/L) additionally received semi-roll spiral compression with an elastic bandage over the dressing for 20 minutes.

**Step 15. Activity restriction.** Movement of the punctured limb was restricted on the day of puncture, and heat application over the puncture site was avoided.

**Step 16. Management of active bleeding.** If active bleeding or oozing occurred, the dressing was changed, the puncture site was re-dressed as in Step 13, and compression was reapplied for another 20 minutes. All events were recorded and graded according to CTCAE v4.03.

# **6. Catheter tip-position verification**

**Step 17. Chest radiography.** Catheter tip position was verified on post-procedural chest X-ray in all patients; an appropriate tip position on chest radiography was required for the placement to be considered successful, consistent with the definition in the main manuscript.

**Note on intracavitary electrocardiographic (IC-ECG) guidance.** An intracavitary electrocardiographic method to confirm tip position, as incorporated in recent PICC protocols for patients with hematologic malignancies [2], was not routinely used in this cohort; tip verification relied on chest radiography. This is stated in the Supplementary Information section of the main manuscript.

# **7. Post-procedural monitoring**

**Step 18. Insertion-site assessment.** The insertion site was assessed at 24-72 hours after catheterization for active oozing, hematoma, redness, swelling, pain, and cord formation (phlebitis).

**Step 19. Thrombosis surveillance.** Venous ultrasound of the catheter-bearing vein was performed whenever catheter-associated thrombosis was suspected clinically; catheter malposition identified on follow-up imaging was corrected by ultrasound-guided repositioning.

**Step 20. Complication documentation.** All catheter-related complications (puncture-site bleeding/oozing, phlebitis, catheter malposition, catheter-associated thrombosis, and catheter-related infection) were documented and graded according to CTCAE v4.03, using the hemorrhage scale, "vascular access complication", "thromboembolic event", and "catheter related infection" scales, respectively.

# **References**

1. Stokowski G, Steele D, Wilson D. The use of ultrasound to improve practice and reduce complication rates in peripherally inserted central catheter insertions: final report of investigation. J Infus Nurs. 2009;32(3):145-55.

2. Picardi M, et al. Thrombotic complications of central venous catheterization with peripherally inserted catheters in patients with classical-Hodgkin lymphoma. Hemasphere. 2025;9(11):e70259. https://doi.org/10.1002/hem3.70259.
